# Brain natriuretic peptide (BNP) may play a major role in risk stratification based on cerebral oxygen saturation by near-infrared spectroscopy in patients undergoing major cardiovascular surgery

**DOI:** 10.1371/journal.pone.0181154

**Published:** 2017-07-12

**Authors:** Hiroshi Mukaida, Masakazu Hayashida, Satoshi Matsushita, Makiko Yamamoto, Atsushi Nakamura, Atsushi Amano

**Affiliations:** 1 Department of Cardiovascular Surgery, Juntendo University Faculty of Medicine, Tokyo, Japan; 2 Department of Clinical Engineering, Juntendo University Hospital, Tokyo, Japan; 3 Department of Anesthesiology & Pain Medicine, Juntendo University Faculty of Medicine, Tokyo, Japan; 4 Department of Clinical Engineering, Kyorin University Faculty of Health Sciences, Tokyo, Japan; Ospedale del Cuore G Pasquinucci Fondazione Toscana Gabriele Monasterio di Massa, ITALY

## Abstract

**Purpose:**

A previous study reported that low baseline cerebral oxygen saturation (ScO_2_) (≤50%) measured with near-infrared spectroscopy was predictive of poor clinical outcomes after cardiac surgery. However, such findings have not been reconfirmed by others. We conducted the current study to evaluate whether the previous findings would be reproducible, and to explore mechanisms underlying the ScO_2_-based outcome prediction.

**Methods:**

We retrospectively investigated 573 consecutive patients, aged 20 to 91 (mean ± standard deviation, 67.1 ± 12.8) years, who underwent major cardiovascular surgery. Preanesthetic baseline ScO_2_, lowest intraoperative ScO_2_, various clinical variables, and hospital mortality were examined.

**Results:**

Bivariate regression analyses revealed that baseline ScO_2_ correlated significantly with plasma brain natriuretic peptide concentration (BNP), hemoglobin concentration (Hgb), estimated glomerular filtration rate (eGFR), and left ventricular ejection fraction (LVEF) (p < 0.0001 for each). Baseline ScO_2_ correlated with BNP in an exponential manner, and BNP was the most significant factor influencing ScO_2_. Logistic regression analyses revealed that baseline and lowest intraoperative ScO_2_ values, but not relative ScO_2_ decrements, were significantly associated with hospital mortality (p < 0.05), independent of the EuroSCORE (p < 0.01). Receiver operating curve analysis of ScO_2_ values and hospital mortality revealed an area under the curve (AUC) of 0.715 (p < 0.01) and a cutoff value of ≤50.5% for the baseline and ScO_2_, and an AUC of 0.718 (p < 0.05) and a cutoff value of ≤35% for the lowest intraoperative ScO_2_. Low baseline ScO_2_ (≤50%) was associated with increases in intubation time, intensive care unit stay, hospital stay, and hospital mortality.

**Conclusion:**

Baseline ScO_2_ was reflective of severity of systemic comorbidities and was predictive of clinical outcomes after major cardiovascular surgery. ScO_2_ correlated most significantly with BNP in an exponential manner, suggesting that BNP plays a major role in the ScO_2_-based outcome prediction.

## Introduction

Tissue oximetry by near-infrared spectroscopy (NIRS) is widely used to monitor cerebral oxygen saturation (ScO_2_) during cardiovascular surgery [1, 2]. Usefulness of intraoperative ScO_2_ monitoring has been reported by many studies [3–12]. However, significance of absolute ScO_2_ values has not been established, since they are influenced by multiple factors such as a composition of focal arterial/venous blood components, oxygen saturation in extra-cerebral tissues, blood hemoglobin concentration (Hgb), and the skull thickness [2, 13–17]. In addition, ScO_2_ values derived from different NIRS devices can differ even within the same subjects [14, 15, 17, 18]. Therefore, ScO_2_ currently is used as a trend monitor rather than as an absolute index of cerebral oxygenation [2]. Intraoperatively, a relative decrease in ScO_2_ from baseline (e.g., 20% decrease) or an absolute threshold ScO_2_ (e.g., <50%) has been used as provisional criteria for cerebral desaturation [1]. However, extremely wide variations in baseline ScO_2_ values raging from less than 20% to more than 80% have been reported [16–18]. Such wide variations seemed unlikely to be explained by aforementioned influencing factors alone. Therefore, it seemed necessary to explore if any factors that might more profoundly influence ScO_2_.

Reportedly, patients with cardiac dysfunction and those with renal failure show lower ScO_2_ than usual [10, 19, 20–23]. In line with these studies, Heringlake et al. showed that ScO_2_ significantly correlated with age, Hgb, N-terminal pro-brain natriuretic peptide (NTproBNP), estimated glomerular filtration rate (eGFR), and left ventricular ejection fraction (LVEF) [24]. ScO_2_ values thus could be associated with risk factors, such as cardiac dysfunction [10, 19, 20, 21, 24], renal dysfunction [21–24], age [24], and anemia [16, 17, 21, 24]. Consequently, Heringlake et al. showed that the baseline ScO_2_ could be predictive of morbidity and mortality after cardiac surgery [24]. However, their findings have not been reconfirmed by other investigators. In addition, although they showed a negative correlation between NTproBNP and ScO_2_, a relationship between brain natriuretic peptide (BNP) and ScO_2_ has not been reported. BNP is an active hormone released from the heart in response to cardiac overloads, whereas NTproBNP is an inactive fragment of precursor proBNP [25]. Because a number of studies showed that compared with NTproBNP, BNP better correlated with indices of cardiac function [26, 27], better detected cardiac dysfunction [28], and better predicted progression of cardiac disease [29], BNP may better correlate with ScO_2_, compared with NTproBNP reported previously [24].

The current study was conducted to examine whether the risk prediction by baseline ScO_2_ values would be reproducible, and to closely characterize the relationship between BNP and ScO_2_, which might contribute to wide inter-individual variations in baseline ScO_2_ values.

## Materials and methods

Prior to the current study, approval was obtained from the Institutional Review Board (IRB) of Juntendo University Hospital. Because of the anonymous and retrospective fashion of the study, the IRB waived the need for patient consent.

### Patients

The current retrospective study included 573 consecutive adult patients, aged 20–91 (mean ± standard deviation, 67.1 ± 12.8) years, who underwent major on-pump or off-pump cardiovascular surgery with ScO_2_ monitoring at Juntendo University Hospital from January 2014 to April 2015.

### Data collection

ScO_2_ was monitored at the bilateral forehead using the INVOS5100C device (Medtronic, Minneapolis, MN). ScO_2_ data were automatically stored every 5–6 seconds in the USB memory stick attached to the device. The baseline ScO_2_ was determined by averaging the bilateral ScO_2_ readings that had been recorded before induction of general anesthesia while patients were breathing room air in a resting position. In addition, the lowest intraoperative ScO_2_ was identified in each patient, and relative decrements in ScO_2_ from baseline were calculated as the maximal drop in ScO_2_ (= the baseline ScO_2_ –the lowest intraoperative ScO_2_) and % maximal drop in ScO_2_ (= the maximal ScO_2_ drop / the baseline ScO_2_ * 100).

Besides demographic variables serving as potential risk factors, the specific cardiovascular risk factors were assessed, including Hgb, BNP, eGFR, LVEF, and the logistic EuroSCORE II as an established risk analysis model [30, 31], using the previous study as a reference [24]. Clinical outcome data included postoperative intubation time, intensive care unit (ICU) stay, hospital stay, postoperative stroke, and hospital mortality.

### Statistical analysis

Because all continuous variables were non-normally distributed after Shapiro-Wilk testing, they are shown as median and quartiles. Categorical data are shown as numbers (%). Because BNP and the EuroSCORE were non-normally distributed in extreme ways, their log-normal transformed values also were used for statistical analyses. Spearman’s correlation coefficient was used to identify factors associated with the baseline ScO_2_. However, Pearson’s correlation coefficient also was used to select candidate variables for multivariate regression analysis, and also to closely illustrate relationships between BNP and the baseline ScO_2_ and that between the EuroSCORE and the baseline ScO_2_. Multiple regression analysis was used to determine factors that could significantly influence the baseline ScO_2_. Bivariate and multivariate logistic regression analyses were used to examine whether the EuroSCORE, absolute ScO_2_ values, and relative ScO_2_ decrements could be predictors of hospital mortality, as described previously [9, 24]. The best cutoff values for significant predictors were further determined by receiver operating characteristic (ROC) analysis, as described previously [9, 24].

Patients were divided into 2 groups based on whether they remained alive or were deceased during hospitalization. In addition, patients were divided into 2 groups also based on whether their baseline ScO_2_ values were ≤50% or >50%, according to the criterion logically set by Heringlake et al [24]. The groups were compared with Mann-Whitney *U* test and Fisher's exact test, as appropriate. A p value < 0.05 was considered significant. Data were analyzed with the software program JMP12 (SAS Institute. Cary, NC).

## Results

### Patients’ characteristics

**Fig 1:**
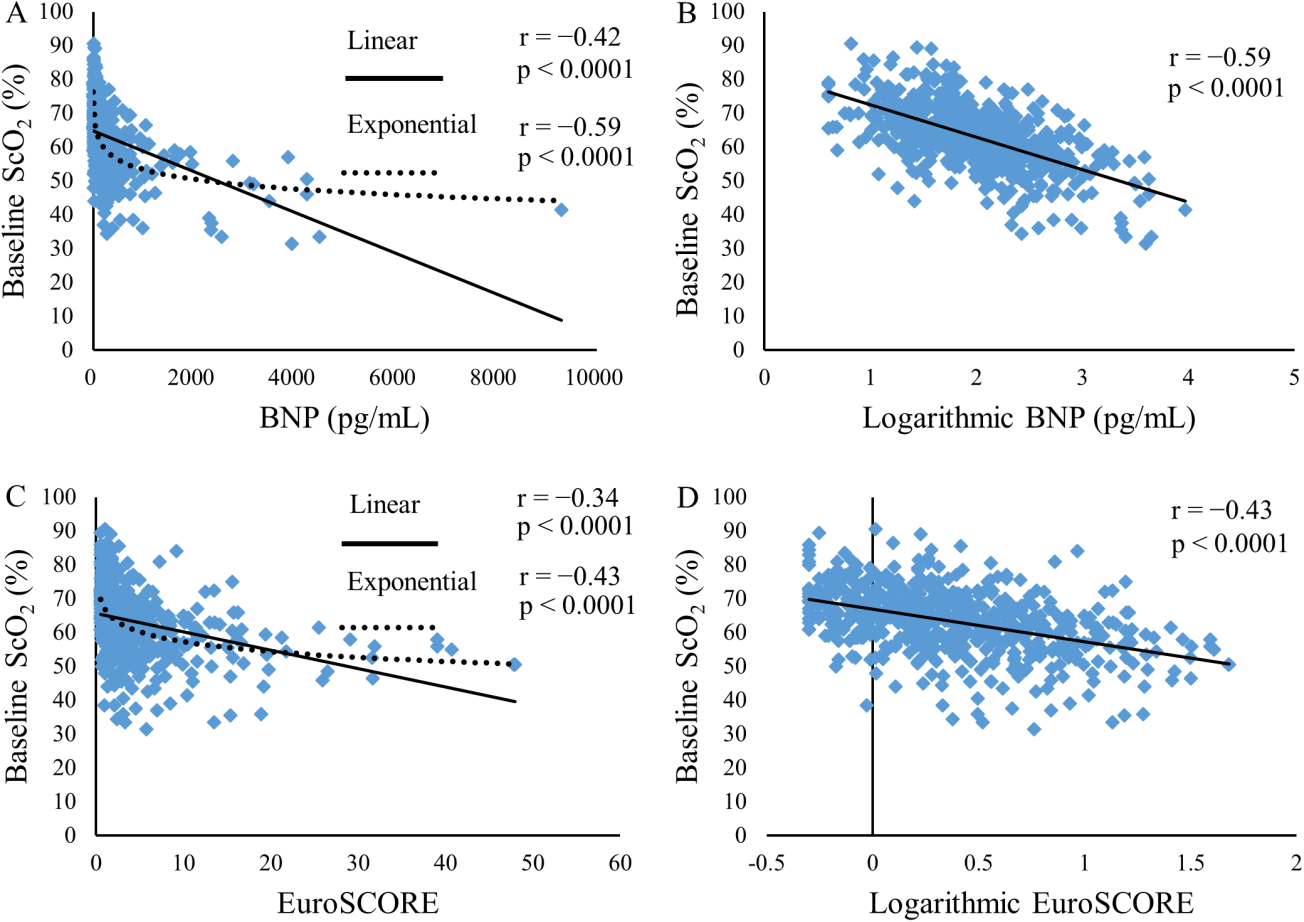
Relationships of BNP, logarithmic BNP, EuroSCORE, and logarithmic EuroSCORE to baseline ScO_2_. Relationships between BNP and baseline ScO_2_ (A), between logarithmic BNP and baseline ScO_2_ (B), between EuroSCORE and baseline ScO_2_ (C), and between logarithmic EuroSCORE and baseline ScO_2_ (D) are shown. Pearson’s correlation coefficients (r) and p values are depicted in each panel. Exponential regression lines, in addition to linear regression lines, are depicted in left panels (A and C). BNP, brain natriuretic peptide; ScO_2_, cerebral oxygen saturation.

**Fig 2:**
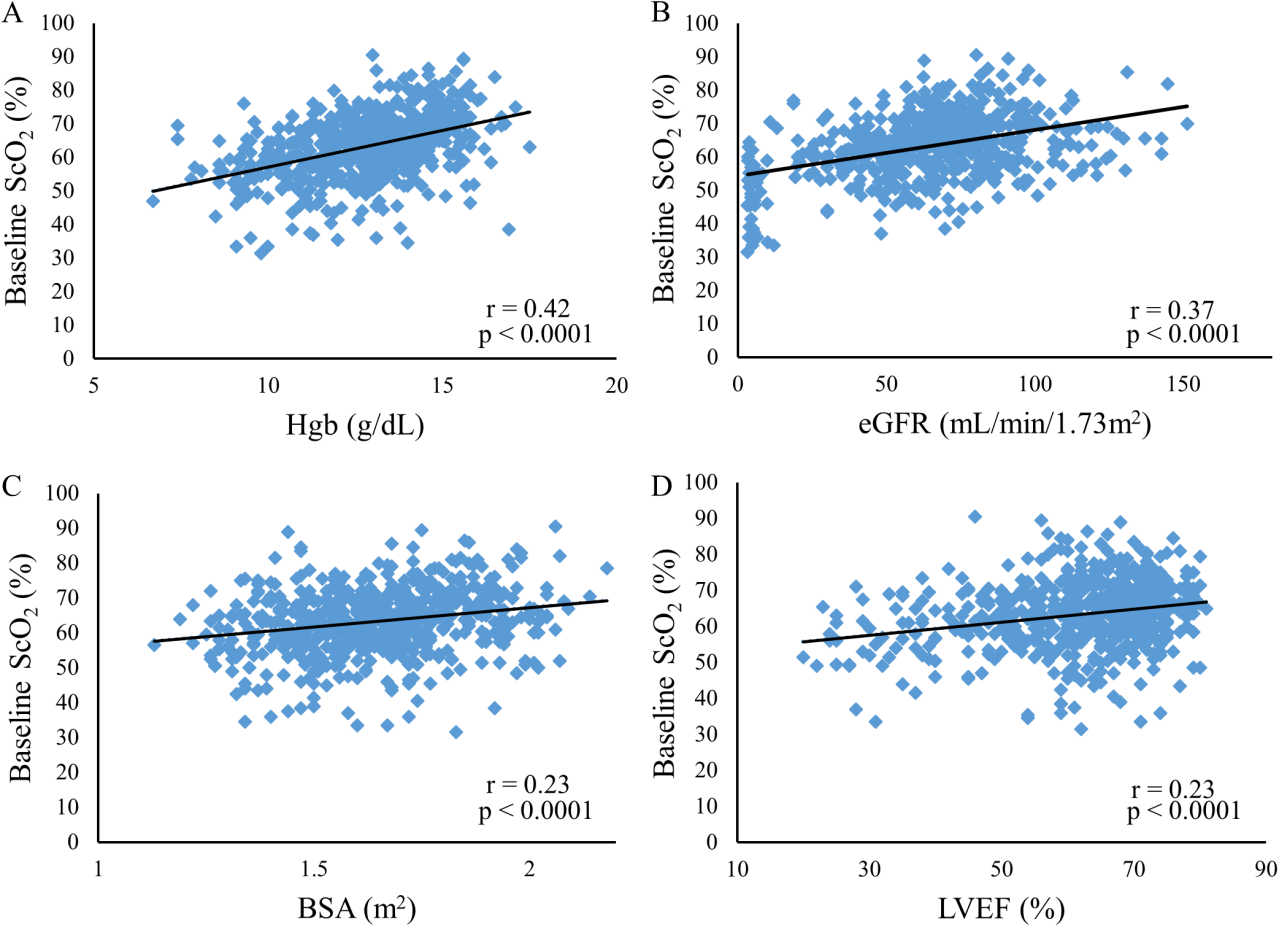
Relationships of Hgb, eGFR, BSA, and LVEF to baseline ScO_2_. Relationships between Hgb and baseline ScO_2_ (A), between eGFR and baseline ScO_2_ (B), between BSA and baseline ScO_2_ (C), and between LVEF and baseline ScO_2_ (D) are shown. Pearson’s correlation coefficient (r) and a p value are depicted in each panel. Hgb, hemoglobin; eGFR, estimate glomerular filtration rate; BSA, body surface area; LVEF, left ventricular ejection fraction; ScO_2_, cerebral oxygen saturation.

Characteristics of the 573 patients in the total cohort are shown in Table 1. Notably, the baseline ScO_2_ before oxygenation and induction of general anesthesia ranged extremely widely from 31.5% to 90.5% (see Figs 1 & 2).

**Table 1:** Patients’ characteristics, surgical procedures, and mortality in 573 patients.

Patients’ characteristics	Total Cohort	Hospital Mortality		
		Alive	Deceased	Significance
	n = 573	n = 561 (97.9%)	n = 12 (2.1%)	
**Demographic data**				
Female	205 (35.8%)	201 (36.5%)	4 (33.3%)	
Age	69 (61–77)	69 (61–77)	74 (71–79)	NS
BSA (m^2^)	1.65 (1.5–1.78)	1.66 (1.5–1.78)	1.6 (1.48–1.68)	NS
**History**				
CKD5D	34 (5.9%)	31 (5.5%)	3 (25%)	p < 0.05
Hypertension	358 (62.4%)	349 (62.2%)	9 (75%)	NS
Dyslipidemia	234 (40.8%)	229 (40.8%)	5 (41.7%)	NS
DM	137 (23.9%)	133 (23.8%)	4 (33.3%)	NS
COPD	57 (9.9%)	57 (9.9%)	0	NS
**Risk stratification**				
NYHA III/IV	53 (9.3%)	50 (8.9%)	3 (25%)	NS
EuroSCORE (%)	2.13 (1.15–4.52)	2.06 (1.14–4.26)	9.79 (5.64–18.3)	p < 0.0001
**Preoperative data**				
BNP (pg/mL)	88.7 (34.3–209.3)	86 (33.4–203.1)	288.5 (59.6–2159)	p < 0.05
Hgb (g/dL)	13 (11.6–14.1)	13 (11.6–14.2)	11.5 (10.7–13.8)	NS
eGFR (mL/min/1.73m^2^)	66.8 (50–81.9)	67.1 (51.2–82.1)	30.5 (14.9–67)	p < 0.01
LVEF (%)	64 (55–70)	64 (55–70)	62 (41.2–71)	NS
Baseline ScO_2_ (%)	63.8 (57.5–69.5)	64 (58–69.5)	54.2 (44.4–62.2)	p < 0.01
**Surgical procedures**				
On-pump CABG	12 (2.1%)	11 (2%)	1 (8.3%)	
CABG + TA replacement	6 (1.1%)	6 (1.1%)	0	
Valve	233 (40.7%)	229 (40.8%)	4 (33.3%)	
Valve + CABG	60 (10.5%)	55 (9.8%)	5 (41.7%)	
Valve + TA replacement	73 (12.7%)	73 (13%)	0	
Valve + CABG + TA replacement	6 (1.1%)	6 (1.1%)	0	
TA replacement	35 (6.1%)	33 (5.9%)	2 (16.7%)	
Myxoma	6 (1.1%)	6 (1.1%)	0	
Adult Congenital	11 (1.9%)	11 (2%)	0	
Off-pump CABG	131 (22.9%)	131 (23.4%)	0	
**Operative course**				
Duration of surgery (min)	236 (174–316)	236 (175–315)	236 (124–401)	NS

Data are expressed as median (quartiles) or numbers (%).

BSA, body surface area; CKD5D, chronic kidney disease, stage 5D; DM, diabetes mellitus; COPD, chronic obstructive pulmonary disease; NYHA, New York Heart Association grading; LVEF, left ventricular ejection fraction; CABG, coronary artery bypass grafting; TA, thoracic aortic; BNP, brain natriuretic peptide; Hgb, hemoglobin; eGFR, estimated glomerular filtration rate; ScO_2_, baseline cerebral oxygen saturation.

### Factors influencing baseline ScO_2_

By both Spearman’s and Pearson’s correlation coefficients, the baseline ScO_2_ correlated highly significantly with logarithmic BNP or BNP, Hgb, eGFR, age, LVEF, and BSA (p < 0.0001 for each) (Table 2). By Pearson’s correlation analysis, the baseline ScO_2_ correlated more closely with logarithmic BNP than with BNP, indicating that the baseline ScO_2_ correlated with BNP in an exponential rather than linear manner (Fig 1A and 1B). On the other hand, the baseline ScO_2_ correlated with Hgb, eGFR, BSA, and LVEF in linear manners (Fig 2). The multiple linear regression analysis revealed that logarithmic BNP, Hgb, eGFR, LVEF, and BSA, but not age, remained significant influencing factors of the baseline ScO_2_, and that logarithmic BNP was the most significant influencing factor (Table 2).

**Table 2:** Results of bivariate and multivariate regression analyses for the baseline ScO_2_.

Variables	Spearman's correlation coefficient		Pearson's correlation coefficient		Standardized partial regression coefficient R^2^ = 0.43 (p < 0.0001)	
	Spearman's ρ	p value	Pearson's r	p value	β	p value
Age	−0.25	p < 0.0001	−0.26	p < 0.0001	0.003	NS
BSA	0.23	p < 0.0001	0.23	p < 0.0001	0.025	p < 0.05
Hgb	0.44	p < 0.0001	0.42	p < 0.0001	0.208	p < 0.0001
Logarithmic BNP	−0.58	p < 0.0001	−0.59	p < 0.0001	−0.417	p < 0.0001
eGFR	0.36	p < 0.0001	0.37	p < 0.0001	0.14	p < 0.0001
LVEF	0.23	p < 0.0001	0.23	p < 0.0001	0.092	p < 0.01

ScO_2_, cerebral oxygen saturation; BSA, body surface area; Hgb, hemoglobin; BNP, brain natriuretic peptide; eGFR, estimate glomerular filtration rate; LVEF, left ventricular ejection fraction.

### Baseline ScO_2_, mortality, and morbidity

Results of the group comparisons between patients alive (n = 561) and deceased (n = 12) are shown in Table 1. In the total cohort, the predicted mortality estimated by the EuroSCORE was 2.13 (1.15–4.52) %, while the actual hospital mortality was 2.09% (12/573) (Table 1). The number of patients with end-stage chronic kidney disease (CKD) was significantly more in deceased patients. The EuroSCORE, and BNP were significantly higher, while eGFR and the baseline ScO_2_ were significantly lower in deceased patients. Age, Hgb, BSA and LVEF were not different between these patients (Table 1).

Results of the group comparisons according to the baseline ScO_2_ are shown in Table 3. Age, BNP, and the EuroSCOR were significantly higher, while BSA, Hgb, eGFR, and LVEF were significantly lower in patients with ScO_2_ ≤50% (n = 528) compared to those with ScO_2_ >50% (n = 45) (Table 3). Postoperative intubation time, ICU stay, and hospital stay were significantly longer, and hospital mortality was significantly higher in patients with ScO_2_ ≤50% compared to those with ScO_2_ >50%, although the incidence of postoperative stroke did not differ between them (Table 3).

**Table 3:** Comparison of risk factors, morbidity, and hospital mortality between 2 groups according to the baseline ScO_2_ values.

	Baseline ScO_2_ ≤50%	Baseline ScO_2_ >50%	p values
	n = 45	n = 528	
Age	74 (66–79)	69 (61–76)	p < 0.05
BSA	1.57 (1.43–1.72)	1.66 (1.51–1.79)	p < 0.05
Hgb	11.5 (9.9–13)	13 (11.7–14.2)	p < 0.0001
BNP (pg/dL)	406.9 (213.2–1767.5)	75.1 (31.8–179.5)	p < 0.0001
eGFR (mL/min/1.73m^2^)	45.2 (5.4–62.2)	69 (53.3–83.3)	p < 0.0001
LVEF%	61 (46–66)	65 (55–70)	p < 0.05
EuroSCORE	5.29 (2.77–10.98)	1.99 (1.1–3.97)	p < 0.0001
Intubation time (h)	8 (5.6–16)	6 (4–10.5)	p < 0.01
ICU stay (days)	4 (2–7.5)	2 (1–3)	p < 0.0001
Postoperative hospital stay (days)	16 (11.5–24)	12 (9–18)	p < 0.001
Postoperative stroke	0 (0%)	15 (2.84%)	NS
Hospital mortality	5 (11.1%)	7 (1.3%)	p < 0.01

Data are expressed as median (quartiles) or numbers (%).

ScO_2_, cerebral oxygen saturation; BSA, body surface area; Hgb, hemoglobin; BNP, brain natriuretic peptide; eGFR, estimated glomerular filtration rate; LVEF, left ventricular ejection fraction; ICU, intensive care unit.

### Prediction of hospital mortality by EuroSCORE, absolute ScO_2_ values, and relative ScO_2_ decrements

Bivariate logistic regression analyses revealed that hospital mortality was significantly associated with the EuroSCORE (p = 0.0005), the baseline ScO_2_ (p = 0.0031), and the lowest intraoperative ScO_2_ (p = 0.018), respectively, but not with the maximal drop in ScO_2_ (p = 0.8928) nor % maximal drop in ScO_2_ (p = 0.5666), indicating that the EuroSCORE and the two absolute ScO_2_ values, but not relative ScO_2_ decrements, could be predictors of hospital mortality. The multivariate logistic regression analysis incorporating the EuroSCORE and the baseline ScO_2_ as independent variables revealed that hospital mortality was significantly associated with both of the EuroSCORE and the baseline ScO_2_ (chi-square 16.3 [p = 0.0003] for overall model fit; odds ratio 1.076 [95% CI, 1.024–1.127; p = 0.0059] for the EuroSCORE; odds ratio 0.937 [95% CI, 0.882–0.997; p = 0.0417] for the baseline ScO_2_). Likewise, the analysis incorporating the EuroSCORE and the lowest intraoperative ScO_2_ revealed that hospital mortality was significantly associated with both of the EuroSCORE and the lowest intraoperative ScO_2_ (chi-square 16.9 [p = 0.0002] for overall model fit; odds ratio 1.097 [95% CI, 1.044–1.150; p = 0.0001] for the EuroSCORE; odds ratio 0.948 [95% CI, 0.903–0.995; p = 0.0275] for the lowest intraoperative ScO_2_). These indicated that each of baseline and lowest intraoperative ScO_2_ values could be predictors of hospital mortality, independent of the EuroSCORE.

### Cutoff values of EuroSCORE, baseline ScO_2_, and lowest intraoperative ScO_2_ for predicting hospital mortality

**Fig 3:**
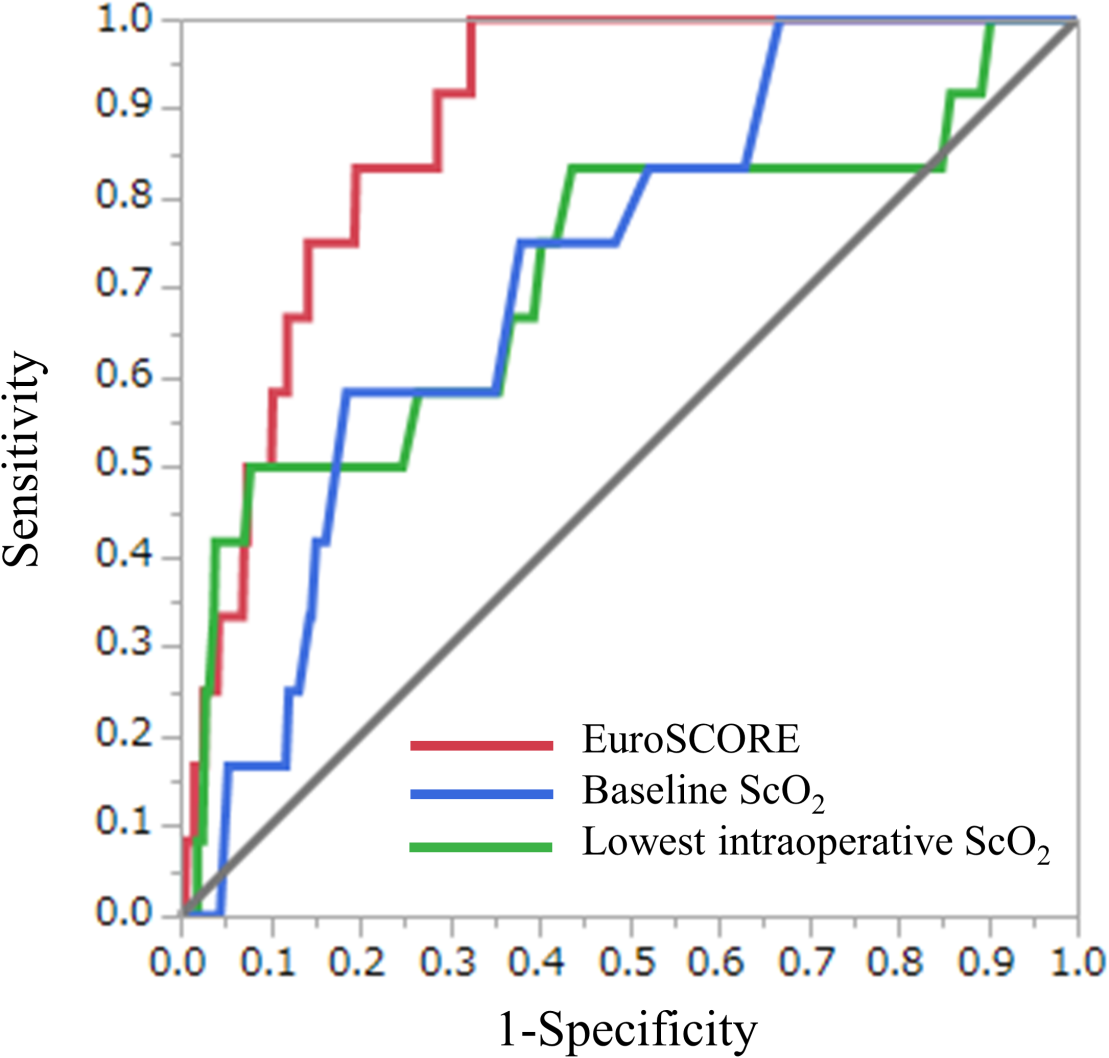
Results of ROC analyses of EuroSCORE, baseline ScO_2_, and lowest intraoperative ScO_2_ for predicting hospital mortality. Areas under curves (AUCs) and p values were 0.883 (95% CI, 0.806–0.932; p < 0.0001) for the EuroSCORE, 0.715 (95% CI, 0.508–0.859; p < 0.01) for the baseline ScO_2_, and 0.718 (95% CI, 0.577–0.826; p = 0.0160) for the lowest intraoperative ScO_2_, respectively. ROC, receiver operating curve; ScO_2_, cerebral oxygen saturation.

ROC analysis of the EuroSCORE and hospital mortality revealed an area under the curve (AUC) of 0.883 (95% CI, 0.806–0.932; p < 0.0001) and a cutoff value of ≥3.25% (sensitivity 100%, specificity 67.5%) (Fig 3). That of the baseline ScO_2_ and hospital mortality revealed an AUC of 0.715 (95% CI, 0.508–0.859; p = 0.0024) and a cutoff value of ≤50.5% (sensitivity 50.0%, specificity 92.2%) (Fig 3). That of the lowest intraoperative ScO_2_ and hospital mortality revealed an AUC of 0.718 (95% CI, 0.577–0.826; p = 0.0160) and a cutoff value of ≤35% (sensitivity 58.3%, specificity 81.5%) (Fig 3). The EuroSCORE tended to have a better accuracy in predicting hospital mortality compared to the baseline ScO_2_ and the lowest intraoperative ScO_2_, but the differences did not reach a statistical significance (differences between areas, 0.168, p = 0.0522; and 0.165, p = 0.0535, respectively).

### Relationship between EuroSCORE and baseline ScO_2_

Similarly to the relationship between BNP and the baseline ScO_2_, the baseline ScO_2_ correlated more closely with the logarithmic EuroSCORE than the EuroSCORE, indicating that ScO_2_ correlated with the EuroSCORE in an exponential rather than linear manner (Fig 1C and 1D). Despite the close correlation between the EuroSCORE and the baseline ScO_2_, both could be independent predictors of hospital mortality, as mentioned above.

## Discussion

### Factors influencing baseline ScO_2_

We found that the baseline ScO_2_ correlated closely with BNP or logarithmic BNP, Hgb, eGFR, LVEF, BSA, and age by bivariate correlation analyses. Previous studies reported that ScO_2_ significantly correlated with Hgb, NTproBNP, eGFR, LVEF, age, and variables associated with body size [16, 17, 20, 21, 24]. To our knowledge, the current study was the first one that demonstrated a significant correlation between BNP and ScO_2_, although Heringlake et al. reported that between NTproBNP and ScO_2_ [24]. Our findings were basically in good agreement with their findings. However, we found a much closer correlation between BNP and ScO_2_ (ρ = −0.58, p < 0.0001) compared to that between Hgb and ScO_2_ (ρ = 0.44, p < 0.0001), in contrast to similar correlation coefficients for Hgb (ρ = 0.37, p < 0.0001) and NTproBNP (ρ = −0.35, p < 0.0001) reported by the previous study [24]. Such a slight difference might result most likely from a difference in patients’ populations studied, but might result also from a difference in peptides examined, since a number of studies showed that compared with NTproBNP, BNP better correlated with cardiac indices [26–29], although some studies reported equal performance of NTproBNP and BNP [32].

In the current study, ScO_2_ correlated with Hgb, eGFR, BSA, and LVEF in linear manners. In contrast, ScO_2_ correlated with BNP in an exponential manner. Possibly, this exponential relationship reflected biologic features of BNP, since previous studies analyzed relationships between BNP and cardiac indices with Pearson’s correlation after log-transforming BNP or with exponential models, indicating that these relationships were better expressed in exponential rather than linear manners [26, 27, 33]. Consequently, we used logarithmic BNP instead of BNP in multiple regression analysis, and found that logarithmic BNP, Hgb, eGFR, LVEF, and BSA, but not age, remained significant factors that were associated with the baseline ScO_2_, and that logarithmic BNP was the most significant factor. BNP was most significantly associated with the baseline ScO_2_ possibly because BNP acted as a surrogate of cardiorenal function that could closely affect baseline ScO_2_ values via its effects on cerebral blood flow and/or cerebrovascular pathology [10, 19, 20, 22, 23]. Our findings also suggested that BNP could be the most significant factor that contributed to the wide inter-individual variations in baseline ScO_2_ values.

### Usefulness of baseline ScO_2_ in risk stratification

As reported previously [24], there was a close correlation between the baseline ScO_2_ and the EuroSCORE. Interestingly, ScO_2_ correlated with the EuroSCORE in an exponential manner. The reason for such a relationship was unclear, but this might be related to the formula for calculating the logistic EuroSCORE, which uses logistic regression analysis incorporating exponential functions in its formula [30].

Because low ScO_2_ values were associated with high BNP, low Hgb, low eGFR, low LVEF, and the high EuroSCORE, low baseline ScO_2_ values might be reflective of severe comorbidities and thus predictive of poor prognosis, as reported previously [24]. Indeed, we found that the baseline ScO_2_ was significantly less in patients deceased than alive. Further, the baseline ScO_2_ ≤50% was associated with increases in intubation time, ICU stay, hospital stay, and hospital mortality. Further, the logistic regression analysis revealed that ScO_2_ could predict hospital mortality independent of the EuroSCORE. The ROC analysis revealed that a cutoff value for the baseline ScO_2_ in predicting hospital mortality was 50.5%, which was very close to the cutoff value of 51% reported previously [24]. As described above, we found the most significant correlation between BNP and ScO_2_. Further, previous studies reported a significant role of BNP in predicting prognosis of cardiac disease [29, 34, 35]. Taken together, it seemed conceivable that BNP played a major role in risk prediction based on the baseline ScO_2_. Heringlake et al. found that a low baseline ScO_2_ value (≤50%) by itself could be predictive of postoperative mortality [24], and we could steadily reconfirm their findings. Hence, it seemed highly likely that cerebral oximetry could have a significant role in risk stratification in patients undergoing cardiovascular surgery. Further, our data suggested that preoperative cerebral oximetry could have an additive value to the EuroSCORE, since the baseline ScO_2_ could be a predictor of hospital mortality independent of the EuroSCORE.

### Significance of absolute ScO_2_ values for outcome prediction

Many studies found links between decrements in ScO_2_ during cardiac surgery and postoperative neurological complications [3–12]. However, in these studies, quite inconsistent criteria for cerebral desaturation were used even using the identical INVOS device [3–12]. Further, most studies had limitations, such as small sample sizes (mostly n ≤100). Therefore, no definite criterion is currently available regarding what threshold ScO_2_ and/or what decrement in ScO_2_ from baseline indicates an abnormal finding during cardiac surgery [1]. However, Schoen et al. revealed, in 231 patients, that the baseline ScO_2_ value, the minimal intraoperative ScO_2_ value, and the AUC below ScO_2_ <50% were associated with postoperative delirium, whereas the relative ScO_2_ decrease or the AUC below 80% of the baseline were not [9]. They reported that cutoff values of the baseline ScO_2_ and the lowest intraoperative ScO_2_ for predicting delirium were 59.5% and 51.0%, respectively [9]. Such results indicated that absolute ScO_2_ values rather than relative ScO_2_ decrements were more relevant in predicting neurological complications. Further, Heringlake et al. showed, in 1178 patients, that patients with baseline ScO_2_ ≤50% were at increased risk for postoperative mortality and those with a preoperative ScO_2_ ≤60% were at increased risk for postoperative morbidity [24]. We also found, in 573 patients, that patients with the baseline ScO_2_ ≤50.5% and the lowest intraoperative ScO_2_ ≤35% were at increased risk for hospital morbidity, and that absolute ScO_2_ values, but not relative ScO_2_ decrements, could be predictors of hospital mortality. Such results indicated that absolute ScO_2_ values rather than relative ScO_2_ decrements were more relevant in predicting postoperative mortality. Hence, low perioperative absolute ScO_2_ might help to identify patients at high risk for postoperative adverse events [5, 6, 8, 12], which highlights the clinical significance of absolute ScO_2_ values. However, further studies in large cohorts are required to identify best cutoff points of perioperative ScO_2_ values for predicting a variety of postoperative complications and mortality.

### Limitations

Our study had several limitations. ScO_2_ was measured only with the INVOS device. Therefore, it remains to be known whether our results would be reproducible with other NIRS devices. Further, as this study was conducted in a retrospective fashion, detailed descriptions of postoperative morbidity were omitted, and there might be any problems with measurement accuracy of ScO_2_ and other variables. Further, although a low baseline ScO_2_ value by itself could be a risk factor for increasing perioperative morbidity and mortality, it remains to be clarified whether low ScO_2_ simply identifies patients with severe comorbidities who are at high risk for postoperative complications or it represents a potentially modifiable risk factor.

## Conclusion

In 573 patients undergoing major cardiovascular surgery, the baseline ScO_2_ correlated with BNP, Hgb, eGFR, and LVEF. BNP was the most significant influencing factor. Further, ScO_2_ correlated with the EuroSCORE. ScO_2_ correlated with Hgb, eGFR, BSA, and LVEF in linear manners, while correlating with BNP and the EuroSCORE in exponential manners. The baseline and lowest intraoperative ScO_2_ values could predict hospital mortality, independent of the EuroSCORE, and the baseline ScO_2_ ≤50.5% and the lowest intraoperative ScO_2_ ≤35% were associated with increased hospital mortality. Low baseline ScO_2_ values were associated with longer needs for postoperative care and higher hospital mortality. The low baseline ScO_2_ was reflective of severity of preoperative systemic comorbidities and was of value for risk stratification in patients undergoing cardiovascular surgery.

## Supporting information

S1 File ‘Available data’. (XLSX)
